# Binding of a Smad4/Ets-1 complex to a novel intragenic regulatory element in exon12 of FPGS underlies decreased gene expression and antifolate resistance in leukemia

**DOI:** 10.18632/oncotarget.2399

**Published:** 2014-08-27

**Authors:** Shachar Raz, Michal Stark, Yehuda G. Assaraf

**Affiliations:** ^1^ The Fred Wyszkowski Cancer Research Laboratory, Department of Biology, Technion-Israel Institute of Technology, Haifa, Israel

**Keywords:** Leukemia, FPGS, Antifolate resistance, Intragenic regulatory element, Smad transcription factors

## Abstract

Polyglutamylation of antifolates catalyzed by *folylpoly-*γ*-glutamate synthetase* (*FPGS*) is essential for their intracellular retention and cytotoxic activity. Hence, loss of FPGS expression and/or function results in lack of antifolate polyglutamylation and drug resistance. Members of the TGF-β/Smad signaling pathway are negative regulators of hematopoiesis and deregulation of this pathway is considered a major contributor to leukemogenesis. Here we show that *FPGS* gene expression is inversely correlated with the binding of a Smad4/Ets-1 complex to exon12 of *FPGS* in both acute lymphoblastic leukemia cells and acute myeloid leukemia blast specimens. We demonstrate that antifolate resistant leukemia cells harbor a heterozygous point mutation in exon12 of *FPGS* which disrupts FPGS activity by abolishing ATP binding, and alters the binding pattern of transcription factors to the genomic region of exon12. This in turn results in the near complete silencing of the wild type allele leading to a 97% loss of FPGS activity. We show that exon12 is a novel intragenic transcriptional regulator, endowed with the ability to drive transcription *in vitro*, and is occupied by transcription factors and chromatin remodeling agents (e.g. Smad4/Ets-1, HP-1 and Brg1) *in vivo*. These findings bear important implications for the rational overcoming of antifolate resistance in leukemia.

## INTRODUCTION

Folates are essential vitamins which serve as cofactors in a variety of key processes such as *de novo* nucleotide biosynthesis, amino acid biosynthesis and DNA methylation [1, 2]. Antifolates are folate antagonists rationally designed to inhibit key enzymes in the folate metabolic pathway and thus disrupt DNA replication [1]. Polyglutamatable antifolates undergo a unique metabolic process known as polyglutamylation, in which several glutamate residues are attached to the γ-carboxyl of the glutamate moiety of the antifolate; this metabolic conversion is catalyzed by the ATP-dependent enzyme folylpoly-γ-glutamate synthetase (FPGS). Polyglutamylation renders antifolates polyanions which, on the one hand, are no longer recognized by efflux transporters [3, 4], and on the other hand, display ~100-fold higher potency to their intracellular target enzyme [2]. Hence, FPGS plays a key role in intracellular retention and antitumor activity of polyglutamatable antifolates [5]. The accumulation of antifolate polyglutamates has been well recognized as an important determinant in the treatment outcome of cancer patients including acute lymphoblastic leukemia (ALL) [6-8] and solid tumors including lung cancer and osteosarcoma [9]. Although antifolates including methotrexate (MTX) are a key component in ALL chemotherapy, acute myeloid leukemia (AML) was found to have intrinsic resistance to these important antimetabolites. Comparison of leukemia blasts obtained from AML patients at daignosis to those derived from ALL patients demonstrates that AML blasts accumulate significantly less long-chain MTX polyglutamates than ALL blasts [10].

We have previously shown that loss of FPGS activity is a predominant mechanism underlying resistance to polyglutamatable antifolates, where 11 out of 14 antifolate-resistant human ALL sublines displayed drug resistance based on impaired FPGS activity [11]. Thus far, three naturally occurring mutations have been shown to underlie loss of FPGS function in leukemia cells: C388F decreased the affinity of FPGS for glutamate by 23-fold [11]. Additionally, C209R and G569C, each identified in separate alleles of *FPGS* in a single antifolate-resistant subline, resulted in ≤13% residual FPGS activity compared to the wild type enzyme [12].

The transforming growth factor-β (TGF-β) signaling pathway has key roles in cell differentiation, apoptosis, development and carcinogenesis [13]. The intracellular effectors of TGF-β signaling are the Sma- and Mad-related (Smad) transcription factors (TFs). While Smad4 is constitutively expressed, it translocates to the nucleus only when in complex with phosphorylated Smads, which are activated by TGF-β (Smad2 and Smad3) or in response to bone morphogenetic proteins (Smad1, Smad5 and Smad8) [14]. In the nucleus, Smads bind directly to their DNA-binding site as heterodimers or interact with various coactivators/repressors [15-18]. TGF-β is considered the most potent negative regulator of hematopoiesis and induces cell cycle arrest in committed progenitors by down-regulating cyclins, cyclin-dependent kinases and c-myc [19] and is considered to have a negative impact on cell proliferation primarily in the myeloid cell lineage [19].

Here we show that Smad proteins are involved in the selective silencing of the WT allele of *FPGS* by binding to an intragenic regulatory element in exon12 of *FPGS* and consequent recruitment of epigenetic modifiers. We further demonstrate that *FPGS* gene expression is inversely correlated with the binding of a Smad4/Ets-1 complex to exon12 in both ALL cells and AML blast specimens.

## RESULTS

### Missense point mutations are a predominant mechanism underlying loss of FPGS activity, leading to resistance to polyglutamatable antifolates in leukemia cells

To explore the mechanisms underlying loss of FPGS function in human T-ALL cells displaying resistance to polyglutamylation-dependent antifolates, we studied the previously described human leukemia antifolate-resistant sublines MTA^R1.5^, MTA C-3 and ZD1694 C-9 [11]. These clonal sublines, which lost over 97% of their cellular FPGS activity consequently displayed high levels of resistance to the polyglutamylation-dependent antifolate ZD1694 (>470-fold compared to parental CCRF-CEM cells), while retaining sensitivity to the non-polyglutamatable antifolate plevitrexed. We first screened the entire *FPGS* coding region for inactivating mutations by cDNA sequencing. Six heterozygous point mutations were identified in these three antifolate-resistant sublines and were mapped to each of the *FPGS* alleles, as detailed in Table 1.

**Table 1:** Characterization of *FPGS* mutations identified in the various antifolate-resistant sublines

Cell line	Point mutation^1^	Amino acid substitution^1^	Location	Conservation	Allele distribution
MTA C-3	G1088A	R363Q	Exon12	100%	Allele A
MTA^R1.5^	G1117C	R356P	Exon12	-	Allele A
	G1185A	A379T	Exon12	100% (A/G)	Allele B
ZD1694 C-9	G699A	V217M	Exon8	-	Allele A
	G909A	E287K	Exon9	80% (D/E)	Allele A
	G1734T	A562S	Exon15	N.A.^2^	Allele B

1 Nucleotides and amino acids were numbered according to the mitochondrial isoform of FPGS, where the first methionine is that of the mitochondrial leader sequence.

2 Not applicable.

**Figure 1:**
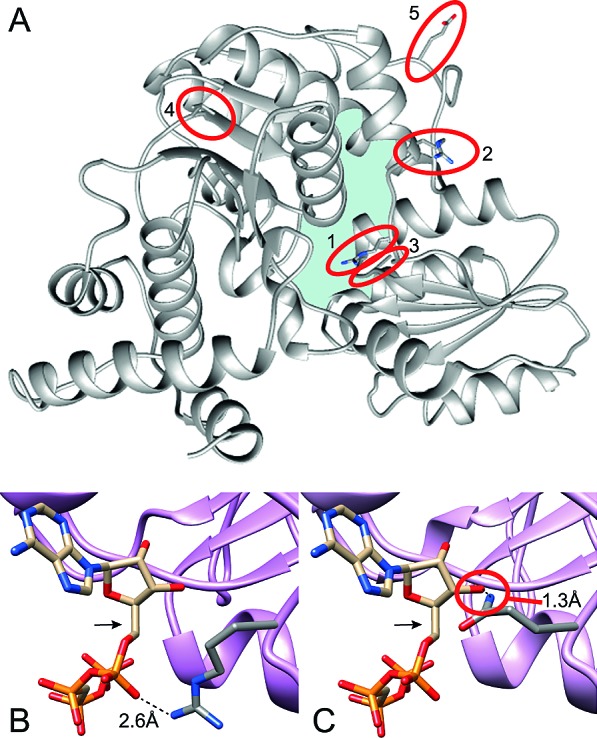
A 3D model of the hFPGS (A) A ribbon diagram of the full hFPGS model, created based upon the crystal structure of the *L.casei* FPGS, with an emphasis on the position of the mutations identified in the current study (marked with red circles). The full side chain of the highlighted residues is presented and colored by element. The mutated amino acids are labeled by red circles and designated as follows: 1. R363, 2. R356, 3. A379, 4. V217 and 5. E287. The ATP-binding pocket is denoted by light blue. A close-up view at the putative interaction of R363 (B) and Q363 (C) with ATP (marked by an arrow) is shown. Distances in Å were determined using the Chimera software.

To explore the possible deleterious effect that these mutations may have on the structure and/or catalytic activity of FPGS, amino acid conservation analysis was performed for the mutated residues by multiple-alignment of the human FPGS (hFPGS) with FPGS from various species ranging from bacteria to mammals (Table 1). Moreover, to further assess the impact of the mutations on protein structure and enzyme function, a 3D model of the hFPGS was built based on the *Lactobacillus casei* (*L. casei*) FPGS template, as detailed in Materials and Methods (Figure 1A). The mutation identified in MTA C-3 cells (i.e. G1088A leading to a R363Q substitution) resulted in the replacement of an arginine residue, which is absolutely conserved among all of species analyzed here, by glutamine. In FPGS from *L. casei*, this arginine residue was shown to form a hydrogen bond with ATP, thus stabilizing it in its binding pocket [20]. Analysis of the R363Q substitution in the 3D model illustrates that while the distance between the native arginine residue and the ATP is compatible with hydrogen bond formation (i.e. 2.6Å, Figure 1B), the mutant glutamine residue faces in the opposite direction, causing a clash with the ATP molecule (Figure 1C). MTA^R1.5^ cells harbor 2 mutations; the first, R356P, affects a non-conserved residue. However, the substitution of the arginine, located in an α-helix at the surface of the protein, with a proline is likely to introduce a bend and thereby have a deleterious effect on protein structure (Figure 1A). The second mutation, A379T, affects a highly conserved small non-polar residue (100% alanine/glycine conservation) which is located at the ATP-binding pocket (Figure 1A). The substitution of an alanine with a polar threonine may cause the formation of a new hydrogen bond or the disruption of hydrophobic interactions, thereby altering protein structure. ZD1694 C-9 cells harbor three mutations; the V217M substitution naturally exists in many species, therefore the substitution of valine with a methionine is not expected to have a deleterious effect on protein structure/function. The allele harboring the V217M mutation harbors an additional E287K substitution that affects a conserved negatively charged residue (80% aspartate/glutamate conservation). However, this residue is located at the protein surface hence the E287K mutation may not affect protein structure or catalytic activity (Figure 1A). The second *FPGS* allele in ZD1694 C-9 cells harbors an A562S substitution which cannot be evaluated by bioinformatics tools since it resides in the C-terminus of the hFPGS - a domain only shared by mammals.

While both MTA^R1.5^ and ZD1694 C-9 sublines harbor missense mutations in both *FPGS* alleles, MTA C-3 cells harbor only a single heterozygous mutation (Table 1) and it was therefore surprising that these antifolate-resistant cells have lost >97% of their cellular FPGS activity. We thus investigated the molecular mechanism underlying the near complete loss of FPGS function in these drug-resistant cells.

### The wild-type *FPGS* allele is selectively silenced in MTA C-3 cells while the expression of the mutant (G1088A) allele is fully retained

**Figure 2:**
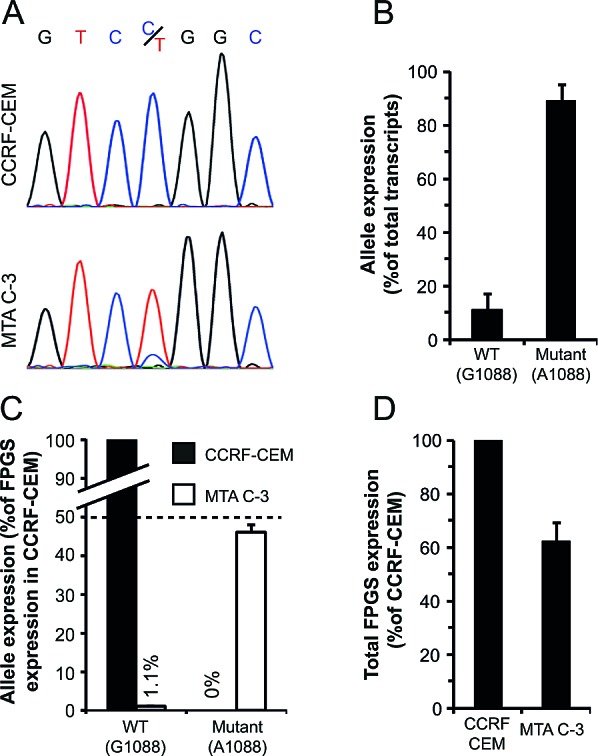
Expression of the WT and mutant alleles of in MTA C-3 cells (A) cDNA sequencing trace of an *FPGS* PCR fragment within exon12 harboring the G/A1088 nucleotide identified in parental CCRF-CEM cells and their antifolate-resistant MTA C-3 subline. (B) Quantification of relative allele expression by TA-cloning analysis. A PCR fragment within exon12 was amplified, using cDNA produced from MTA C-3 cells, and cloned, followed by sequencing of individual clones. The results are the means of three independent experiments performed on different RNA extractions ± S.D. In each experiment, at least 20 clones were sequenced. (C) Quantification of allele expression by allele specific real-time PCR analysis performed on RNA extracted from MTA C-3 cells and compared to that from parental CCRF CEM cells. (D) Total *FPGS* transcript expression in CCRF-CEM cells and their antifolate-resistant subline MTA C-3 determined by real-time PCR using primers residing within the second exon. The results are the means of at least three independent experiments, each performed in triplicates ± S.D.

The cDNA sequencing trace of exon12 in MTA C-3 cells revealed a markedly higher representation of the mutant A1088 allele over the WT G1088 allele, represented by the height of the nucleotide peak which could not be detected in parental CCRF-CEM cells (Fig 2A). To corroborate this finding, relative allele expression was determined by TA-cloning of a cDNA PCR product spanning a region containing the G1088A mutation, followed by sequencing of individual cDNA clones. This analysis of multiple *FPGS* cDNA clones revealed that the WT allele constitutes only 10% of all *FPGS* transcripts (n=60), whereas the remaining 90% of *FPGS* transcripts harbored the G1088A mutation (Fig. 2B). To assess whether the WT allele is silenced or, alternatively, the mutant allele is overexpressed, we quantified their expression by real-time PCR using allele-specific primers, designed to diagnostically discern between the WT and mutant *FPGS* alleles. The expression levels of the WT and mutant alleles in MTA C-3 cells were compared to the expression of the WT allele in parental CCRF-CEM cells (transcribed from both alleles of the *FPGS* gene) by real-time PCR analysis. If both the WT and mutant alleles in MTA C-3 are fully transcribed, each allele should yield 50% of the expression of *FPGS* in CCRF-CEM; this analysis revealed that while the mutant allele is fully transcribed (46±2%, Fig. 2C), the WT allele is completely silenced (1.1±0.2%, Fig. 2C). Selective silencing of the WT allele should result in a 50% decrease in total *FPGS* mRNA levels, therefore we determined *FPGS* expression in MTA C-3 cells compared to their parental counterpart; indeed, total *FPGS* mRNA levels in MTA C-3 cells were reduced by ~40% (Figure 2D). These results establish that the WT *FPGS* allele is exclusively silenced in MTA C-3 cells.

Alterations in the expression levels or post-translational modifications of *trans*-acting elements could not account for this differential allele-specific silencing since they should equally affect the WT and mutant *FPGS* alleles. Hence, to explore the mechanism underlying differential *FPGS* allele expression in MTA C-3 cells, we first searched for sequence alterations in transcriptional and post-transcriptional *cis*-regulatory elements (i.e. promoter and 3′-UTR). In this respect, Freemantle and Moran previously showed that the minimal promoter (MP) of *FPGS* resides 43bp upstream to the first methionine of the mitochondrial leader sequence and extends 150bp into the first exon [21]. We therefore sequenced a 300bp DNA fragment upstream to the first methionine, the entire exon1 as well as the entire 3′-UTR; we found that the MP of *FPGS* was free of mutations, whereas the 3′-UTR contained the single nucleotide polymorphism ss1509426 (T1956C) in both parental and MTA C-3 cells. Thus, no sequence alterations in these regulatory elements could account for the differences in the expression of the WT and mutant *FPGS* alleles in MTA C-3 cells.

### Exon12 of *FPGS* has transcriptional regulatory capability

**Figure 3:**
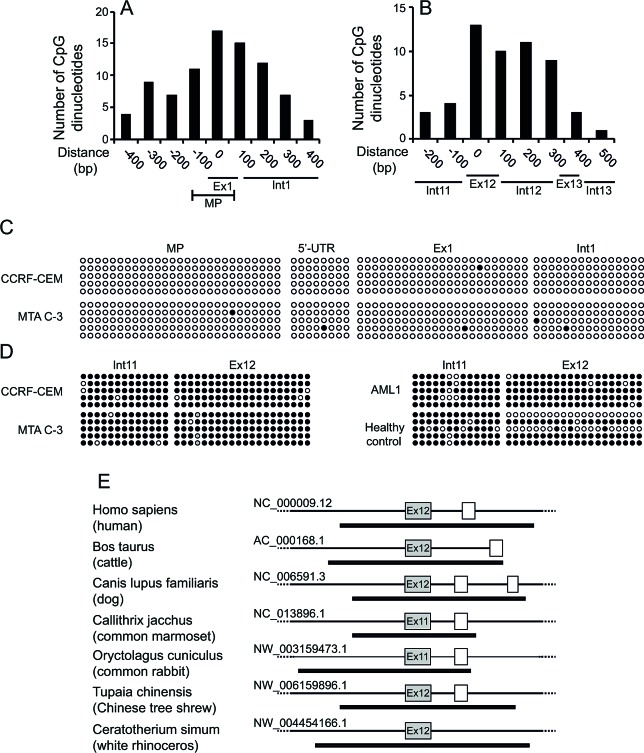
Prediction of CpG island and methylation analysis within the *FPGS* gene (A, B) CpG dinucleotide content within the predicted putative CpG islands in the *FPGS* promoter (A) and exon12 (B), was assessed by the Methyl Primer software. MP, minimal promoter. The X-axis denotes the distance from the transcription start site, for the MP, or from the int11-exon12 border, for exon12. (C, D) Actual status of CpG island methylation in the minimal promoter (C) and exon12 of *FPGS* (D) in parental CCRF-CEM cells, their antifolate-resistant MTA C-3 subline, an AML patient and a healthy control, as determined by bisulfite-based DNA sequencing. Each line represents a single clone, whereas each row represents a different CpG dinucleotide. Solid and open circles represent methylated and unmethylated CpG dinucleotides, respectively. In MTA C-3 cells, the G1088A substitution results in the loss of one CpG dinucleotide, which is represented by grey circles. (E) Prediction of the intragenic CpG islands encompassing exon12 of *FPGS* in various mammals. Boxes represent exons, whereas lines represent introns. Exons homologous to exon12 of the *hFPGS* are colored in grey. The predicted CpG islands are marked by a thick black line.

Recently it was shown that treatment of cells with histone deacetylase inhibitors such as suberanilohydroxamic acid (SAHA) increases the intracellular accumulation of long-chain MTX polyglutamates [22]. Hence, we postulated that epigenetic alterations, including DNA methylation and repressive histone modifications, may possibly account for the selective silencing of the WT allele in MTA C-3 cells. To explore this hypothesis, we screened the *FPGS* gene for CpG islands and found two such elements: one encompassing the promoter region (Figure 3A) and another spanning the entire surroundings of exon12 (Figure 3B). To explore whether or not allele-specific methylation in the *FPGS* promoter or exon12 can account for the selective silencing of the WT allele in MTA C-3 cells, the methylation status of these regions was assessed by bisulfite sequencing analysis. We found that the promoter of *FPGS* is completely unmethylated (Figure 3C), whereas heavy methylation was found from intron 11 throughout exon12 in both the WT and mutant alleles in MTA C-3 and CCRF-CEM cells (Figure 3D). The G1088A mutation, found within the CpG island, disrupts a CpG dinucleotide, hence enabling us to verify that both alleles were represented during the cloning and sequencing in the bisulfite sequencing analysis of exon12 (Figure 3D). We confirmed that this methylation is not limited to cell lines by determining the methylation status of exon12 in leukemic cells from an adult AML patient and white blood cells from a healthy individual. Indeed, exon12 was found to be methylated in human cells as well (Figure 3D).

Recent studies have shown that not only promoters but also intragenic and intergenic regions are widely modulated during various physiological processes and diseases. In particular, it is becoming increasingly clear that DNA methylation in the gene body is not just a passive witness of gene transcription but it appears to be actively involved in multiple gene regulation processes [23]. Moreover, intragenic CpG island methylation was recently shown to mark transcriptional regulatory elements, especially when the CpG island is evolutionarily conserved [24]. Therefore, we performed an alignment of the hFPGS with various FPGS proteins from different mammals to assess which exons are homologous to human exon12, and then determined the presence of a putative CpG island in the genomic region of these homologues. A putative CpG island (highlighted by a dark black line) was found to encompass the exons that are homologous to the human exon12 among various mammals including cattle, dog, common marmoset, rabbit, tree-shrew and rhinoceros (Figure 3E).

**Figure 4:**
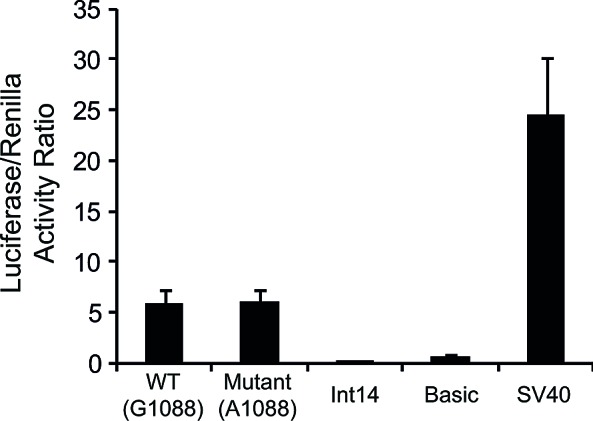
Luciferase reporter gene assay The promoter activity of the WT and mutant exon12 of *FPGS* was determined by luciferase reporter gene analysis, and was compared to the activity of the SV-40 promoter, the luciferase vector without a promoter (Basic) or to a 150bp DNA fragment from intron14 of *FPGS*. Results shown are the ratio of the Luciferase to Renilla readings, and are the means of three independent experiments performed in duplicates ± S.D.

Given that our results suggest a transcriptional regulatory role for exon12 of *FPGS*, we determined the ability of the WT and mutant exon12 to initiate and actively drive transcription of a luciferase reporter gene. The sequences of the WT and mutant exon12 were cloned and served as the promoters of the luciferase reporter gene and luciferase expression driven by these exons was compared to: 1) a random 150bp fragment from *FPGS* intron 14, 2) a luciferase vector lacking a promoter (i.e. pGL3-basic) as well as 3) an SV-40 promoter. The WT and mutant exon12 displayed as much as 24% of the promoter activity of the SV-40 promoter, whereas the 150bp fragment from intron14 of *FPGS* had no promoter activity, being comparable to the promoterless vector (Fig. 4).

### The WT allele of FPGS is selectively bound by transcription factors and epigenetic modifying agents at the exon12 region

**Figure 5:**
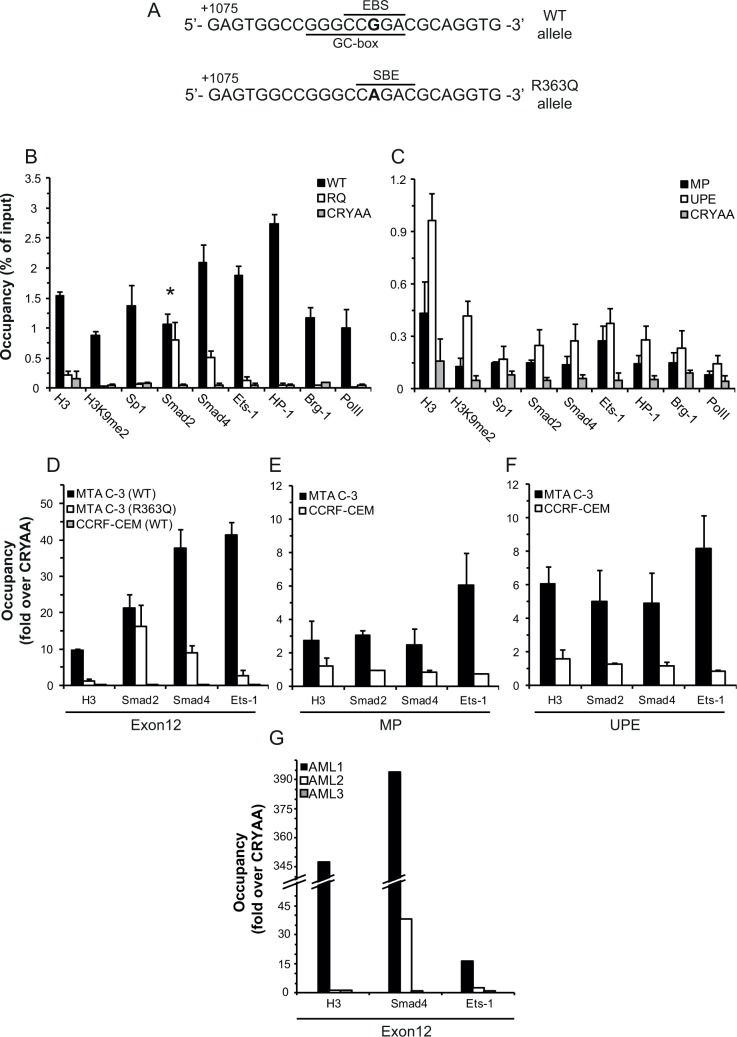
ChIP analysis of the binding of various factors to different regions of the *FPGS* gene (A) Bioinformatics prediction of biding sites for TFs in the WT and G1088A mutant exon12 using MatInspector. The G/A1088 nucleotide is in bold. EBS-Ets binding site, GC-box-Sp1 binding site, and SBE-Smad binding element. The nucleotide position is relative to the first ATG. (B) ChIP analysis of the protein occupancy at the WT and mutant exon12 of *FPGS* compared to the methylated *CRYAA* in MTA C-3 cells. *P*-values for the differential allele binding ≤0.05 except for Smad2, marked by an asterisk. (C) ChIP analysis of the protein occupancy at the minimal promoter (MP) of *FPGS* or the upstream promoter element (UPE) compared to the methylated *CRYAA* in MTA C-3 cells. *P*-values ≤0.05 except for H3, Ets-1, Brg-1 and PolII at the MP, as well as Sp1 and Brg-1 at the UPE. (D-F) ChIP analysis of the binding of histone H3, Ets-1, Smad2 and Smad4 to exon12 (D), to the MP (E) or to the UPE (F) of *FPGS* in CCRF-CEM and MTA C-3 cells compared to the binding to *CRYAA* in these cells. (G) ChIP analysis of the binding of H3, Smad4 and Ets-1 to exon12 of *FPGS* in 3 AML patients. (D-G) The results are represented as fold enrichment over the binding to *CRYAA*. All ChIP experiments, except for those undertaken with patient blood samples, were performed at least 3 times, and the results are the means of at least three real-time PCR analyses conducted in triplicates ± SD.

The sole difference we observed between the WT and the mutant R363Q allele in MTA C-3 cells was the G1088A mutation, which resides in exon12. This exon can effectively drive gene transcription (Figure 4), hence we hypothesized that the *cis/trans*-regulatory elements responsible for differential allele expression reside within exon12. We thus analyzed by bioinformatics the mutation region for the loss or *de novo* formation of binding sites for TFs. This analysis revealed that WT exon12 harbors binding sites for both avian erythroblastosis virus E26 (v-ets) oncogene homolog-1 (Ets-1) (EBS, Figure 5A) and specificity protein 1 (Sp1) (GC-box, Figure 5A). In contrast, whereas these binding sites are disrupted by the mutation, a known consensus sequence for the binding of TFs of the Smad family is newly formed (SBE, Figure 5A). Therefore, we determined the actual binding of Ets-1, Sp1, Smad2 and Smad4 (Smad 3 is not expressed in these cells and thus was not used) to the WT and mutant alleles by chromatin immunoprecipitation (ChIP) analysis, followed by allele specific real-time PCR. Expectedly, this ChIP analysis revealed that Ets-1 and Sp1 bind to the WT allele 15- and 20-fold over the mutant allele, respectively (representing 1.4 and 1.9% of the input, respectively; Fig. 5B). Furthermore, Smad4, which is known to interact with Ets-1 [15], was also found to differentially bind the WT allele (4-fold over the mutant allele, representing 2.1% of the input in the WT allele), whereas Smad2 was found to bind both alleles equally (Figure 5B). Smad proteins were previously shown to be important factors in promoting and maintaining epigenetic gene silencing [18, 25, 26] and have also been suggested to be involved in the recruitment of heterochromatin protein 1 (HP-1), a known marker of gene repression, by promoting histone H3K9 methylation [18]. Therefore, we further determined, by ChIP analysis, the binding of the following epigenetic markers of gene silencing to exon12 of *FPGS*: 1) Brahma-related gene 1 (Brg-1) as a marker of the nucleosome positioning complex SWItch/Sucrose Non-Fermentable (SWI/SNF) in addition to histone H3 and histone H3K9me2 modification as markers of high nucleosome density and repressive histone modifications; 2) HP-1α as a marker of heterochromatin and gene repression; and 3) RNA polymerase II (PolII), as a marker of RNA polymerase stalling/pausing due to high nucleosome abundance [27, 28]. Indeed, Brg-1, HP-1α, and PolII exhibited >25-fold higher abundance on the WT allele compared to the mutant allele, and H3 had a >7-fold higher abundance on the WT allele (Figure 5B). Moreover, at the WT allele we found H3K9me2 to constitute 54% of the detected histone H3, whereas at the mutant allele only 14% of the detected histone H3 was found to harbor this epigenetic modification. The *αA-crystallin* (*CRYAA*) promoter is known to be methylated in most cells [29]; we hence used the *CRYAA* gene as a negative control for protein occupancy in our ChIP experiments. Protein binding to the mutant allele of *FPGS* in MTA C-3 cells resembled the binding to *CRYAA* (i.e. residual ~0.1%), with the exception of Smad2 and Smad4, which bind the mutant allele 9-16 fold over *CRYAA* (Figure 5B).

Exon12 is ~7kb downstream to the *FPGS* promoter, suggesting that it may affect allele expression by DNA looping with the MP of *FPGS*. We hence expected to find in the promoter the same binding of the TFs and epigenetic modifiers which were found in the WT *FPGS* allele, although most do not have a binding site in the promoter sequence. While exon12 of *FPGS* was highly occupied by TFs, the MP of *FPGS* had 7-20 fold lower binding of these proteins to give a maximum input percentage of 0.27 (Figure 5C). Since the MP did not exhibit substantial protein binding as would be expected from a promoter, we searched for additional regulatory elements. In this respect, Leclerc *et al* (2006) showed that a crucial *FPGS* promoter element resides ~2500bp upstream of the first methionine in CCRF-CEM cells [30]. Therefore, the binding of these proteins to the upstream promoter element (UPE) was evaluated by ChIP analysis. Indeed, the same binding pattern was detected in this promoter region compared to the binding to exon12, with higher occupancy than in the MP (Figure 5C). In contrast to MTA C-3 cells, CCRF-CEM cells fully express the *FPGS* gene. We therefore further postulated that the binding of TFs to exon12 in CCRF-CEM cells should resemble the binding to the mutant exon12, with the exception of Smad2/4. ChIP analysis confirmed that there is only residual protein binding at exon12 of *FPGS* in CCRF-CEM cells (signal to noise ratio ≤1, Fig. 5D). Consistently, the binding of these proteins to the MP of *FPGS* or to the UPE in CCRF-CEM was also undetected (<1.5-fold over background, Fig. 5E-F).

To explore the clinical relevance of our findings, which suggested that the expression of *FPGS* is repressed by a Smad4/Ets-1 complex, we studied the binding of these TFs to exon12 of *FPGS* in blast cells from AML patients. We first determined *FPGS* expression in white blood cells from adult AML patients at diagnosis (N=9) compared to healthy volunteers (N=7). We found that most of the AML patient blast specimens (N=7) expressed increased *FPGS* mRNA levels (≥200% relative to healthy controls), whereas 2 patient specimens, designated AML1 and AML2, displayed substantially low levels of *FPGS* (i.e. 40% and 25% of control *FPGS* expression, respectively) (Table 2). We therefore performed ChIP analysis to determine the actual binding of H3, Smad4 and Ets-1 to exon12 of FPGS in AML1 and AML2 patient specimens, compared to an AML3 patient sample which expressed high levels of *FPGS* (208% of healthy controls). Strikingly, leukemic blast cells from both AML1 and AML2 patient specimens exhibited extremely high abundance of Smad4 at the genomic region of *FPGS* exon12, representing 71% and 4.8% of the input, with a signal to noise ratio of 394 and 38, respectively (Figure 5G). This was in sharp contrast to the binding of Smad4 to exon12 in AML3 patient specimen, which displayed only a negligible binding representing 0.8% of the input with a signal to noise ratio of 1. The binding of Smad4 in AML1 and AML2 patient blasts was accompanied with Ets-1, though to a lesser extent, representing 4.3% and 1.7% of the input, respectively, with a signal to noise ratio of 16 and 2.7 (Figure 5G). Consistently, the signal to noise ratio of the binding of Ets-1 to exon12 in AML3 patient specimen was 1. In addition, the binding of Smad4/Ets-1 was highly correlated with the presence of H3 in the region of exon12 in AML1 patient specimen with a signal to noise ratio of 350 (Figure 5G).

**Table 2:** AML Patients' characteristics at diagnosis

Patient	Age	Sex	WBC/μl	Percent blasts	*FPGS* expression^1^
AML1	57	Female	23000	70	39
AML2	75	Male	23400	18	25
AML3	52	Female	203600	85	208
AML4	19	Female	32200	75	180
AML5	62	Male	14750	55	1109
AML6	21	Female	40840	80	182
AML7	52	Female	23600	84	244
AML8	70	Female	20000	62	250
AML9	69	Male	40000	50	361

1 Percent of *FPGS* expression in white blood cells obtained from healthy donors.

## DISCUSSION

In the current study we explored the molecular mechanism underlying antifolate resistance in leukemia cells harboring a heterozygous G1088A mutation in *FPGS*. We found that the WT *FPGS* allele was selectively silenced presumably due to the binding of Smad4 to the genomic region of WT exon12. We further show that *FPGS* expression is inversely correlated with the binding of Smad4 to exon12 in leukemic blast cells from adult AML patient specimens.

Our search for the mechanism underlying the selective silencing of the WT allele of *FPGS* in MTA-C3 cells led us to discover a heavily methylated CpG island in the region encompassing intron 11 through exon12 of the *FPGS* gene in human leukemia cell lines and in human peripheral white blood cell samples (i.e. healthy donor and an AML patient). In addition, bioinformatics analysis revealed that exon12 harbors a putative GC-box sequence, which we found to be occupied by the TF Sp1 (17-fold over the negative control) upon ChIP analysis. Although the functional role of intragenic binding of TFs has not been extensively reported, Sp1 and Smads were previously shown to regulate transcription via intragenic binding sites [31, 32]. These findings suggested that exon12 of *FPGS* has a transcriptional regulatory role. A careful examination of the UCSC database revealed several points to support our hypothesis that exon12 acts as a regulator of *FPGS* transcription: 1) Exon12 emerges upon DNaseI hypersensitivity assays along with the MP of *FPGS*; indeed, DNase I hypersensitivity is an established feature of regulatory elements [33]. 2) Histone modifications which are characteristic of promoter/enhancer elements including H3K4me3 and H3K4ac are observed in exon12 in various tumor cell lines. 3) A high occupancy of TFs in exon12 was reported in ChIP-seq experiments compared to other regions of the *FPGS* gene.

In this respect, since our findings indicate that exon12 may play an important role in regulating *FPGS* gene expression, we performed ChIP analysis to confirm the actual binding of TFs to exon12 or to the MP of *FPGS*. The consensus site for Smads is present only in the mutant *FPGS* allele, which could explain the binding of Smad2 and Smad4 to the mutant allele in MTA C-3 cells demonstrated by ChIP analysis. However, we found that Smad2 and Smad4 bind the WT *FPGS* as well. Smads were shown to have low binding affinity to DNA, thus requiring additional cofactors for DNA binding [15], and are known to interact with Sp1 [17] and Ets-1 [15]. Indeed, the latter TFs were selectively bound to the WT allele, thus providing a plausible explanation for the preferential binding of Smad4 to the WT allele over the mutant allele. Additionally, genome-wide analysis of Smads binding established that Smad4 is found in regions distant from the transcription start site and in GC-rich motifs [34], both of which are characteristics of the exon12 region of *FPGS*.

In the current study we found that Smad4 can bind the WT *FPGS* allele *in vivo* and recruit epigenetic modifying proteins, leading to the near complete silencing of the WT *FPGS* allele. We further demonstrate the binding of Smad4 and Ets-1 to the genomic region of exon12 of *FPGS* in AML patient blasts exhibiting low *FPGS* expression, while no such binding was detected in an AML patient specimen with high *FPGS* levels. Thus, the current study establishes Smads as novel negative regulators of *FPGS* gene expression. This implies that a combination regimen consisting of both antifolates along with TGF-β inhibitors in the treatment of leukemia may have a substantial synergistic therapeutic impact on patient outcome. Consistently, the TGF-β signaling pathway has been successfully targeted in hepatocellular carcinoma cells and triple-negative breast cancer cells with a small molecular inhibitor (LY2157299) or with a humanized neutralizing antibody against TGF-β receptor type II [35, 36]. Such targeted combination chemotherapy may improve the treatment efficacy of both ALL and AML via two approaches: 1) ALL patients could be tested for Smad4-binding to exon12 of FPGS at diagnosis and/or upon relapse, thereby allowing clinicians to personalize the treatment protocol for individual ALL patients. 2) It may enable the inclusion of antifolates in the treatment of AML, a hematological malignancy which is known to be inherently resistant to antifolate treatment due to low levels of FPGS activity [10]. FPGS is crucial for the maintenance of the intracellular folate pool, which is critical for *de novo* nucleotide biosynthesis and DNA replication [1], thus it is not surprising that we found *FPGS* to be overexpressed in most of the specimens from adult AML patients (7 out of 9 patients studied). This is in concordance with previous reports in which *FPGS* was found to be overexpressed in many hematopoietic malignancies, and to a lower extent in normal bone marrow [37]. From a physiological perspective, we propose that part of the TGF-β response, which induces cell cycle arrest in hematopoietic cells, leads to silencing of *FPGS* expression via binding of Ets-1, Smad2 and Smad4 to exon12 of *FPGS*, thereby resulting in reduced intracellular nucleotide pools.

DNA-looping between distant regulatory elements within the ORF and the promoter is known to contribute to transcriptional regulation [38], hence, exon12 of *FPGS* may interact with the promoter, with the cooperation of the highly bound Smad4/Ets-1 complex. This Smad4/Ets-1 complex can also promote allele-specific silencing by recruiting epigenetic modifiers. However, whereas these TFs highly bind exon12, they were found at a much lesser extent in the MP (~2% of input in exon12 compared to ~0.3% of input in the MP). The promoter of *FPGS* was previously explored in CCRF-CEM leukemia cells by Leclerc *et al*, with constructs harboring promoter segments up to 8kb upstream of the transcriptional start site, revealing an 800bp domain located ~2.5kb upstream of the transcription start site with an important regulatory role [30]. We thus evaluated Ets-1, Smad2 and Smad4 occupancy in this upstream region, and indeed found a significant abundance of these TFs in the UPE. Bioinformatics analysis identified binding sites for these factors in the UPE, allowing for this genomic region to interact with exon12 by cross-talk between the same TFs. This is further substantiated by the fact that in CCRF-CEM parental cells Ets-1 and Smads do not bind to exon12 and accordingly, the MP and UPE are also devoid of these proteins. The putative interaction of exon12 with the promoter presumably directs the epigenetic modifiers to allow specific allele silencing. The SWI/SNF complex can either promote transcription by nucleosome removal or suppress transcription by nucleosome positioning, depending on the interacting protein partners (reviewed by Narlikar et al, 2002 [39]). This complex was shown to interact with methyl CpG binding proteins to repress gene expression [40] and since exon12 is highly methylated, it may recruit the *trans*-acting factors necessary for transcriptional repression. High nucleosome density impairs the unwinding of the two DNA strands during transcription and was shown to induce PolII stalling as well as pausing [27, 28]. We consistently found here 50-fold higher levels of PolII occupancy on the WT allele, when compared to the mutant allele. Another support to the epigenetic silencing of the WT allele is that the sequences of both WT and mutant alleles had the same promoter activities in driving luciferase reporter transcription. Since luciferase reporter analysis is performed using a vector transiently introduced into the cells, nucleosomes cannot be recruited and cannot influence gene expression. Therefore, if indeed epigenetic modifications are the mechanism underlying silencing of the WT allele, no differences in luciferase reporter activity are expected between the WT and mutant alleles.

In this study we also demonstrate that inactivating *FPGS* mutations constitute a dominant mechanism of antifolate resistance in leukemia cells, illustrated by the fact that all antifolate-resistant cells that were examined harbored inactivating mutations in the ORF of *FPGS*. We have previously shown (Liani *et al*., 2003) that loss of FPGS activity is a dominant mechanism of resistance to polyglutamatable-antifolates in leukemia cells and our conclusion was that inactivating mutations are not a common mechanism of loss of FPGS function in antifolate resistance [11]. However, the screening for inactivating mutations at that time was performed by the single-strand conformational polymorphism (SSCP) method, which is based on conformational changes induced by single-base mutations. Clearly, since various mutations may not necessarily introduce a conformational change in the DNA fragment under study, this SSCP method is not sufficiently sensitive as the direct sequencing method in detecting mutations. Hence, we were now able to detect multiple single nucleotide substitutions in the *FPGS* gene in various antifolate resistant leukemia sublines. In this respect, we identified a total of six missense mutations in the coding region of *FPGS*, which further expand the list of *hFPGS* mutations including three other missense mutations previously reported to result in the loss of FPGS activity in antifolate resistant cells [11, 12]. Two of the mutations that we identified here, in two independent antifolate-resistant sublines, affected the ATP-binding pocket of FPGS. The R363Q substitution found in MTA C-3 cells is predicted to impair ATP-binding by interfering with an important hydrogen bond formed between the conserved arginine residue at position 363 and ATP. The A379T substitution found in MTA^R1.5^ cells resides at close vicinity to the ATP-binding pocket and may disrupt substrate entry or release from this pocket. The A562S substitution, found in MTA^R1.5^ cells, could not be analyzed using bioinformatics tools. However, substitution of the G569 residue located near the A562 residue, with a cysteine, resulted in the loss of 87% of FPGS activity in antifolate resistant human leukemia cells [12], strongly indicating that this region is highly important for FPGS activity. Polymorphisms affecting FPGS protein activity have recently been reported in the general population, suggesting different antifolate sensitivities in patients undergoing antifolate-containing chemotherapy [41]. Since we used single-step selection to isolate two of the antifolate resistant clonal sublines, the mutations that we identified were most likely preexisting variants rather than newly induced by the exposure to antifolates. Therefore, we suggest that in addition to the variations in FPGS activity between patients, the tumor itself, in a single patient, is composed of multiple genomically unstable cells which may display distinct sensitivities to antifolates due to high frequency of alterations in the coding sequence of the *FPGS* gene. Some of these cells can survive the chemotherapeutic treatment and can result in a relapse of the disease. Recent studies support this notion hence establishing the striking intratumor genomic heterogeneity when analyzing multiple regions of the same tumor in individual cancer patients [42].

In summary, we found that in addition to loss of FPGS enzyme activity, single nucleotide substitutions in *FPGS* can result in alterations in allele expression. This is due to a unique transcriptional regulatory role of the downstream exon12 discovered herein, which is a binding site for a Smad4/Ets-1 complex. The current study constitutes the first report of the impact of the TGF-β/Smad signaling pathway on *FPGS* gene expression. These novel findings have important ramifications for the rational overcoming of FPGS-based antifolate resistance in leukemia.

## MATERIALS AND METHODS

### Chemicals

Antifolates were obtained from various sources as previously described [43].

### Cell culture

Human leukemia cell lines were maintained as previously described [11]. MTA C-3, MTA^R1.5^ and ZD1694 C-9 cells were generated from parental T-cell leukemia CCRF-CEM cells as previously described [11]. Briefly, MTA C-3 and ZD1694 C-9 cells were established using a single step exposure to 460nM MTA or 70nM ZD1694 (~20-fold the antifolate concentration required to achieve 50% growth inhibition in parental CCRF-CEM cells), respectively, followed by clonal isolation using the limiting dilution method. MTA^R1.5^ cells were generated by multiple step selection initiated at 25nM MTA and terminated at a concentration of 1.5μM.

### Patient samples

Analysis on AML samples was performed on blast cells obtained from adult AML patients at diagnosis at the Department of Hematology Oncology, Rambam Medical Center, Haifa, Israel. The samples were previously derived as part of the routine clinical management and were used in the current study after receiving approval from the local institutional review board (study no. 2902) at the Rambam Medical Center and informed consent in accordance with the Declaration of Helsinki. White blood cells were isolated from peripheral blood of patients or healthy controls by a standard Ficoll-Hypaque (Sigma, St. Louis, MO, USA) gradient density centrifugation. The cells were frozen in aliquots in fetal calf serum supplemented with10% DMSO until analysis.

### DNA sequencing and real-time PCR analysis

Cells (5×10^6^) from the mid-log phase of growth were harvested and total RNA was isolated using the TRI Reagent kit (Sigma, St. Louis, MO, USA). cDNA synthesis was carried out using the High-Capacity cDNA Reverse Transcription Kit (Applied Biosystems-Life Technologies, Grand Island, NY, USA) according to the manufacturer's instructions and PCR was performed using ReddyMix PCR Mastermix (Thermo scientific, Waltham, MA, USA). Sequencing was performed using a 3500xL Genetic Analyzer for Resequencing & Fragment Analysis (Applied Biosystems-Life Technologies). The primers used for DNA sequencing are depicted in Table 3.

**Table 3:** Primers used in various applications

Open reading frame and promoter sequencing			Tm=60°C	
FPGS 1	Fw	CCGGGCCTAGAGCGCTG	Rv	TTCCCCTTCGTCCCAGTGAC
FPGS 2	Fw	CCGGCTGAACATCATCCA	Rv	AGCATCGGACACAGGTATAGA
FPGS 3	Fw	TCTCCTCTCTTGGCATCGA	Rv	CGGTCCCCGGTAGCATTGA
FPGS 4	Fw	AGGCCTGCGTGCGCTGGTT	Rv	AGGCAGCGCACACAATAAGC
FPGS pr	Fw	GCTTTTTAGTGGCGCAAGG	Rv	GTCTCCGAATTCCCAGCCC
**Real-time PCR analysis**			**Tm=60°C, ^1^Tm=70°C**	
Exon12 WT (cDNA)^1^	Fw	ACGGAGTGGCCGGGCCGG	Rv	CCTCTCGCGGCCCTGCAGC
Exon12 WT (ChIP)^1^	Fw	ACGGAGTGGCCGGGCCGG	Rv	CCTGCAGCGCCTGGCGGAA
Exon12 RQ (cDNA)^1^	Fw	CACGGAGTGGCCGGGCCA	Rv	AGACTCGAACCTCGGGGCCACC
Exon12 RQ (ChIP)^1^	Fw	CACGGAGTGGCCGGGCCA	Rv	TGCAGCGCCTGGCGGAACC
FPGS RT (cDNA)	Fw	CCGAGCATGGAGTACCAGGA	Rv	GCGCTTCACCTGCTCCAG
β2M (cDNA)	Fw	GGCTATCCAGCGTACTCCAAA	Rv	CGGCAGGCATACTCATCTTTTT
MP (ChIP)	Fw	CTGCCCCACCATCCCCA	Rv	CGCCCCAAACTACCACCG
UPE (ChIP)	Fw	TGGGACACATACTTAGTCGTCA	Rv	CAGAGCAAGACTTTGTCTCGA
CRYAA (ChIP)	Fw	TCCACCATCAGCCCCTACTA	Rv	GTTGCATCTTACCTCAGAGA
**Bisulfite analysis**			**Tm=62°C, ^2^Tm=60°C**	
Bis MP1	Fw	AATGTTGGGAAGAGGGAGAGG	Rv	CRCCCCCAACCAATCAAC
Bis MP1A (Nested, on MP1)	Fw	AATGTTGGGAAGAGGGAGAGG	Rv	AACAACCCTCAATCTCTACCCC
Bis MP1B (Nested, on MP1)	Fw	GGGTAGAGATTGAGGGTTGTTG	Rv	CRCCCCCAACCAATCAAC
Bis MP2	Fw	GTTGATTGGTTGGGGGYG	Rv	TTCTAAAACCCTCCACTACCCC
Bis MP 2A (Nested, on MP2)	Fw	GTTGATTGGTTGGGGGYG	Rv	CCTAATCCCTATCCCCACCC
Bis MP 2B (Nested, on MP2)	Fw	GGTGGGGATAGGGATTAGGAAG	Rv	TTCTAAAACCCTCCACTACCCC
Bis ex12	Fw	TAAGTTTTTTTAGTAAATGGTGGG	Rv	AACCAACCCCRACCCCAC
Bis ex12 A^2^(Nested, on ex12)	Fw	TAAGTTTTTTTAGTAAATGGTGGG	Rv	CCCTACCCCTCACCCRCTC

Real-time PCR was performed using an Applied Biosystems-Life Technologies 7300 Real-Time PCR. Quantitative PCR reaction (20μl) contained: 5ng of cDNA, 150nM of primers and 1x PerfeCTa qPCR FastMix, UNG, ROX (Quanta Biosciences, Gaithersburg, MD, USA). The primers used for quantification of *FPGS* gene expression were FPGS RT Fw and Rv. The primers used for allelic quantification in MTA C-3 cells were exon12 WT Fw and Rv and exon12 RQ Fw and Rv for the detection of the WT and mutant alleles, respectively. Allele-specific PCR was performed at a high Tm (70°C), and allele and gene expression levels were normalized to *β-2-microglobulin* (*β2M*) which was used as an internal control. To compare the expression levels of the WT and mutant alleles of FPGS in MTA C-3 cells to the expression of the WT allele in CCRF-CEM cells, we first performed a real-time PCR on known template amounts and compared the threshold cycle (Ct) values obtained with each primer. We found that the primers detecting the WT allele reached the Ct value one cycle earlier than the primers detecting the RQ allele. We then calculated the delta-Ct for each sample by subtracting the Ct of the *β2M* gene from the WT/mutant allele Ct. To compensate for the differences in primers Ct values, we added one cycle to the delta Ct values obtained with the WT allele primer. The results were then calculated relative to the expression of the WT allele in CCRF-CEM cells based on the 2^−ΔΔCt^ method.

The primer sequences used for real-time PCR are depicted in Table 3. Each experiment was performed independently at least three times, and all real-time PCR experiments were performed in triplicates.

Nucleotides and amino acids were numbered according to the mitochondrial isoform of FPGS, where the first methionine is that of the mitochondrial leader sequence.

### Bioinformatics analyses

For the analysis of amino acid conservation, Jalview was used to visualize the multiple alignment generated by psi-blast and to calculate conservation of each residue.

To generate the 3D model of the hFPGS, a multiple alignment of the hFPGS was performed by 10 iterations of HHpred using HHblitz MSA generation method. The *L.casei* FPGS, the crystal structure of which was previously reported (PDB 1jbw), scored the highest in conservation and was therefore used as a template to generate the 3D model of the hFPGS using Modeller. The model was visualized using UCSF Chimera [44].

MatInspector (Genomatix) was used to analyze the MP, UPE and exon12 of *FPGS* for putative TFs binding sites.

Putative CpG island predictions in the *FPGS* gene of the indicated species were performed by Methyl primer express (Applied Biosystems-Life Technologies) with the following parameters: 300-2,000bp CpG island length, over 50% GC content and CpG observed/CpG expected ratio >0.6. A multiple alignment was generated as described above, and was used to identify the location of the putative CpG island relative to the *hFPGS* exon12 homologue in each species.

### Cloning analysis

To determine relative allele expression in MTA C-3 cells, a PCR reaction was performed using the primers FPGS 3 Fw and FPGS 3 Rv, spanning the region of the mutation. The PCR product was purified using Wizard® SV Gel and PCR Clean-Up System (Promega, Fitchburg, WI, USA) and cloned using the pGEM T-easy vector systems (Promega). Plasmids from single clones were extracted using GeneJET Plasmid Miniprep Kit (Thermo Scientific) and sequenced as described above. Three experiments were performed on separate RNA extractions; in each experiment at least 20 clones were sequenced.

### DNA methylation analysis

To assess DNA methylation in the CpG islands, genomic DNA was isolated using The DNeasy Blood & Tissue Kit (QIAGEN, Hilden, Germany). Following DNA bisulfite conversion using the EpiTect Bisulfite Kit (QIAGEN), bisulfite-treated DNA was amplified in two subsequent rounds of PCR using primers designed by the Methyl Primer Express software v1.0 (Applied Biosystems-Life Technologies). The primer sequences are depicted in Table 3. PCR products were cloned and 5 clones were sequenced as described in the supplementary methods.

### Luciferase reporter gene assay

For the cloning of the WT and mutant exon12 of *FPGS* into pGL3-basic luciferase reporter vector (Promega), WT exon12 was amplified from CCRF-CEM cDNA and the mutant exon12 was amplified from MTA C-3 cDNA using the following primers: NheI/Exon12 Fw (5′-TAATGCTAGCTTCGGAACACGGAGTG) and Ex12/BglII Rv primer (5′-TAATAGATCTCGGCCTCTCGCGG). PCR products were purified using Wizard® SV Gel and PCR Clean-Up System (Promega) and cut along with the pGL3-basic vector using the restriction enzymes NheI and BglII (NEB). For the cloning of a 150bp fragment from intron 14, PCR was performed on genomic DNA from MTA C-3 using the primers SmaI/intron14 Fw (5′-ATCTCCACCTCCCGGGTTC) and Int14/DpnII Rv (5′-GGCGGATCACCTGAGGTCAA). The PCR product was first resolved on a 2% agarose gel and purified using Wizard® SV Gel and PCR Clean-Up System (Promega), and then it was excised with SmaI and DpnII (NEB, Ipswich, MA, USA). Ligations were performed using DNA ligation kit (Takara, Otsu, Shiga, Japan) according to the manufacturer's instructions.

For the luciferase assay, MTA C-3 cells were electroporated with 10μg of each vector together with 0.2μg of pRLO-Renilla vector (0.234kV). Thirty-six hours following transfection, luciferase activity was determined using the Dual-Luciferase® Reporter Assay System (Promega) according to the manufacturer's instructions on a GloMax 20/20 luminometer (Promega).

### Chromatin Immunoprecipitation (ChIP) analysis

ChIP analysis on patient specimens and cell lines was performed by harvesting 1-2×10^6^ cells for each IP and fixation with 1% formaldehyde for 15 min. Cross-linking was quenched with 125mM glycine for 5 min and fixed cells were washed twice with ice-cold PBS. Cells were then lysed with buffer A (5mM PIPES, pH 8.0, 85mM KCl and 0.5% Nonidet P-40) for 10 min on ice. The fraction of crude nuclei was sedimented by microcentrifugation (5000rpm, 5min, 4°C), washed with Nonidet P-40-free buffer A, resuspended in buffer B (10mM EDTA, 50mM Tris-HCl, pH 8.1, and 1% SDS), and incubated on ice for 10 min. The fraction of crude nuclei was sonicated on ice ten times for 10s each with 10s intervals (Misonix Microson, amplitude 3), followed by centrifugation (16,000×g, 10min, 4°C). A 1/10 aliquot was preserved as an input sample. The supernatant was diluted 10-fold in immunoprecipitation (IP) buffer (16.7mM Tris, pH 8.1, 167mM NaCl, 1.2mM EDTA, 0.01% SDS, 1.1% Triton X-100). Pre-clearing was performed by incubation of the diluted protein-DNA samples with 10μl of MagnaBind protein G beads (30min, 4°C; Thermo Scientific) to eliminate unspecific adsorption to the beads. Samples were then incubated with antibodies against histone H3 (2μg), H3K9me2 (2μg), Sp1 (4μg), Smad2 (4μg), Smad4 (4μg), Ets-1 (4μg), HP-1α (4μg), Brg-1 (4μg) and PolII (2μg) (overnight, 4°C). Histone H3 and H3K9me2 antibodies were from Abcam (Cambridge, MA, USA), HP-1α antibody was from Millipore (Billerica, MA, USA), other antibodies were from Santa Cruz Biotechnology (Dallas, TX, USA). Immune complexes were allowed to form by slow mixing on a rotating platform at 4°C overnight. To collect immune complexes, samples were incubated with 20μl of MagnaBind protein G beads (2h, 4°C), following which 5 consecutive washes were performed (1ml wash, 5min each) with the following buffers: 1) low salt buffer (20mM Tris-HCl, pH 8.1, 2mM EDTA, 150mM NaCl, 0.1% SDS, 1% Triton X-100); 2) high salt buffer (the same buffer except for the inclusion of 300mM NaCl); 3) LiCl buffer (10mM Tris-HCl, pH 8.1, 0.25M LiCl, 1mM EDTA, 1% Nonidet P-40 and 1% deoxycholate); 4) TE buffer (10mM Tris-HCl, 1mM EDTA, pH 8.1); the last wash was repeated twice. Immune complexes were eluted twice by the addition of 100μl of elution buffer (0.1M NaHCO_3_, 1% SDS) and incubated for 15 min with rotation mixing at room temperature. To reverse cross-linking, the samples and input were incubated at 65°C for 2h in 200mM NaCl. Next, 40mM Tris-Cl (pH 6.5), 10mM EDTA, and 20μg of pronase were added to each sample which was then incubated at 42°C for 45 min. DNA was isolated from each sample using the DNA Wizard® SV Gel and PCR Clean-Up System (Promega). All buffers were supplemented with protease inhibitors (complete mini, EDTA-free protease inhibitor mixture tablet, Roche Applied Science, Indianapolis, IN, USA).

Analysis of protein occupancy was performed using real-time PCR. Occupancy was calculated as percent of input for each of the genomic regions which were analyzed. When occupancy was presented as percent of *CRYAA*, we first determined the percent of input for exon12, MP and UPE and then divided the results with the percent of input for the *CRYAA* gene, obtained for each antibody. The primer sequences used for real-time PCR are depicted in Table 3.

### Statistical Analyses

We used a 1-tailed unpaired Student's t-test to examine the significance of the difference between the control and treatment groups (N≥3). A difference was considered significant if the P value obtained was < 0.05.
